# *In vitro* immunomodulation of magnesium on monocytic cell toward anti-inflammatory macrophages

**DOI:** 10.1093/rb/rbaa010

**Published:** 2020-04-14

**Authors:** Lei Sun, Xiaoyu Li, Menghan Xu, Fenghe Yang, Wei Wang, Xufeng Niu

**Affiliations:** r1 Key Laboratory for Biomechanics and Mechanobiology of Ministry of Education, School of Biological Science and Medical Engineering, Beihang University, No. 37 XueYuan Road, Haidian District, Beijing 100083, China; r2 Beijing Advanced Innovation Center for Biomedical Engineering, Beihang University, No. 37 XueYuan Road, Haidian District, Beijing 100083, China; r3 Department of Immunology, School of Basic Medical Sciences, NHC Key Laboratory of Medical Immunology, Peking University, No. 38 XueYuan Road, Haidian District, Beijing 100191, China

**Keywords:** immunomodulation, magnesium, THP-1, macrophage, polarization

## Abstract

Biodegradable magnesium (Mg) has shown great potential advantages over current bone fixation devices and vascular scaffold technologies; however, there are few reports on the immunomodulation of corrosive Mg products, the micron-sized Mg particles (MgMPs). Human monocytic leukemia cell line THP-1 was set as the *in vitro* cell model to estimate the immunomodulation of MgMPs on cell proliferation, apoptosis, polarization and inflammatory reaction. Our results indicated high-concentration of Mg^2+^ demoted the proliferation of the THP-1 cells and, especially, THP-1-derived macrophages, which was a potential factor that could affect cell function, but meanwhile, cell apoptosis was almost not affected by Mg^2+^. In particular, the inflammation regulatory effects of MgMPs were investigated. Macrophages exposed to Mg^2+^ exhibited down-regulated expressions of M1 subtype markers and secretions of pro-inflammatory cytokines, up-regulated expression of M2 subtype marker and secretion of anti-inflammatory cytokine. These results indicated Mg^2+^ could convert macrophages from M0 to M2 phenotype, and the bioeffects of MgMPs on human inflammatory cells were most likely due to the Mg^2+^-induced NF-κB activation reduction. Together, our results proved Mg^2+^ could be used as a new anti-inflammatory agent to suppress inflammation in clinical applications, which may provide new ideas for studying the immunomodulation of Mg-based implants on human immune system.

## Introduction 

Biomaterials have been commonly applied in chronic diseases and clinical surgeries in recent years, e.g. joint replacement implants for total hip arthroplasty [1], bioresorbable vascular scaffolds for acute myocardial infarction [2], hemostatic sponge for surgical wound hemostasis [3] or dermal matrix for skin transplantation [4]. However, considerable implant failures take place following the surgical procedure of implantation, which limit their further applications [5]. Inflammation is considered one of the major reasons for implantation failure, which occurs in early stages after implantation, and the activation of inflammatory cells plays a crucial role in this process. Inflammatory cells can respond to biomaterials by identifying the relevant properties of material (e.g. degradability, surface chemistry and topography) and regulate the microenvironment surrounding biomaterials by releasing cytokines, chemokines and other factors to affect the tissue regeneration. Therefore, the inflammatory response is tightly regulated to initiate the healing process facilitating tissue repair.

In the biomaterial-regulated reactions, the host immune response is divided into the following stages: blood–biomaterial interaction, inflammation, foreign body reactions (FBRs) and fibrous capsule formation [6]. Among all kinds of inflammatory cells, macrophages and monocytes play important parts in inflammation and FBR triggered by biomaterial implantation [7–10]. In brief, when circulating monocytes derived from committed progenitor cells in bone marrow migrate to peripheral blood, they can differentiate into monocyte-derived macrophages to participate in immune response [8]. In particular, uncommitted macrophages (M0) are highly plastic cells, which can exhibit a spectrum of polarization states in response to different environmental cues. At one end of the spectrum, there is the ‘classically activated’ pro-inflammatory macrophages (M1) and at the other end ‘alternatively activated’ anti-inflammatory macrophages (M2) state [7, 11–13]. M1 phenotype has typical surface markers CD86 and CCR7, produces interleukin-1β (IL-1β) and tumor necrosis factor-α (TNF-α) cytokines and enhances T helper 1 cell-mediated inflammation [14], while M2 phenotype has the typical surface markers CD206 and CD163, secretes IL-10 and transforming growth factor-β cytokines, enhances T helper 2 cell-mediated inflammation, relieves inflammation and improves tissue repair and regeneration [7].

Mg is the second most abundant divalent cation within the cellular systems. This essential element has a variety of biological functions in regulating energy metabolism, enzyme activity, signal transduction, nucleic acid and protein synthesis. For instance, magnesium sulfate (MgSO_4_) can be used to treat pre-eclampsia and preterm birth, reducing the risk of pediatric cerebral palsy [15]. Moreover, Mg has been considered as the most promising metal for biomedical material applications and other clinical procedures based on their unique biodegradability, biocompatibility, good mechanical properties and osteogenesis ability [16–20]. The previous research revealed that Mg has better mechanical strength and degradation rate when it is alloyed with various elements and presents successful osseous tissue regeneration after implantation [21, 22]. In addition, unlike other permanent metallic implants, Mg implants do not cause stress shielding, chronic local inflammation and permanent physical stimulation. However, Mg-based biomaterials have their own problems. When degraded in the body, Mg is corroded rapidly and induces hydrogen gas (H_2_) cavities formation [23–25]. The excessive biocorrosion rate leads to the local accumulation of Mg in the body, which may cause pathophysiological and toxicological changes, such as hypermagnesemia. This complication can increase the risk of death by affecting the respiratory and cardiovascular systems [26, 27]. Therefore, the evaluation on the safe concentration range of Mg^2+^ is necessary, especially in monocytes and macrophages. In addition to H_2_, another byproduct from rapid biocorrosion of Mg or Mg alloys is micron-sized Mg particles (MgMPs) [18, 28]. The previous studies showed the ions and wear debrises released from metallic implants could cause serious inflammation in the microenvironment surrounding the implants, leading to the implantation failure [29, 30]. Recent researches suggested that Mg alloy does not induce systemic inflammation or adverse impacts on cellular function [31, 32], but a clear and systematic understanding of the inflammatory effects for Mg^2+^ on human inflammatory cells is still lacking, especially on the polarization behavior of macrophage.

In present research, we studied the immunomodulation of Mg^2+^ released from MgMPs on inflammatory cells *in vitro*, so as to provide new insights into clinical applications of biomaterials. Based on the highly similarity to primary monocytes and macrophages in biology, THP-1 human cell line and its derived macrophage were used in the present study [33].

## Methods and materials

### MgMPs samples and extract preparation

MgMPs (average diameter 31.02 μm, >99% purity) were purchased from Tangshan Weihao Magnesium Powder Co., Ltd. (Qian'an, China) and analyzed by scanning electron microscope (SEM, FEI Quanta 250 FEG, USA). After UV sterilization for 1 h, excessive MgMPs (0.5 g/ml) were soaked in serum-free RPMI 1640 (Gibco, USA) for 1 week (at 37°C, under 5% CO_2_), to prepare MgMPs extracts. After that, the mixture was centrifuged and the supernatants were harvested. Then, MgMPs extracts were buffered to the pH 7.4 by hydrochloric acid (HCl) and diluted into different concentrations by RPMI 1640 to study the Mg^2+^ concentration-dependent effects. The Mg^2+^ concentration and pH value in collected extracts were determined by inductively coupled plasma atomic emission spectrometer (ICP, PerkinElmer Optima 5300DV, USA) and pH meter (Leici PHS-3C, China), respectively.

### Cell culture and differentiation

THP-1 cells were obtained from the American Type Culture Collection (ATCC, USA) and maintained at 2 ∼ 10 × 10^5^/ml in RPMI 1640 supplemented with 10% fetal bovine serum (FBS, Gibco, USA) in a humidified chamber under 5% CO_2_ at 37°C. The cells were passaged every 2 days and only early passage cells (p3–5) were used in the subsequent study.

For phorbol ester (PMA)-induced differentiation, 50 ng/ml PMA (Sigma-Aldrich, USA) were dispensed in cell culture medium. After cocultivation for 2 days, the cells treated with PMA were differentiated into THP-1-derived macrophages. Cells (PMA treated or not) cultured with RPMI 1640 medium and MgMPs extracts of different Mg^2+^ concentrations containing 10% FBS were set as the control and test groups, respectively. In subsequent experiments, all the cells were seeded into 12-well plates (Eppendorf, Germany) at 1.5 × 10^5^/ml for 1, 3 and 6 days under the culture environment mentioned above.

### Proliferation and cytomorphology test

In order to evaluate the influence of Mg^2+^ on cell proliferation, THP-1 cells or THP-1-derived macrophages were cultured in MgMPs extracts with different Mg^2+^ concentrations (m-1, m-1/2, m-1/4 and m-1/8) for 1, 3 and 6 days. Then, 100 µl cell suspension from each group and 10 µl CCK-8 (Dojindo, Japan) were added to each well of a 96-well plate. After 3 h of incubation, the absorbance was detected by the microplate reader (ThermoFisher, USA) at 450 nm. The proliferation of cells was calculated as follows: RGR (cells relative growth rate) = (OD test)/(OD control) × 100%. The corresponding cell morphology was observed using the microscope (Olympus, Japan).

### Flow cytometry analysis

#### Cell apoptosis

The apoptotic induction effect of MgMPs extracts on THP-1 cells and THP-1-derived macrophages was determined by using the apoptotic kit (Roche, Switzerland). In brief, cells treated with the MgMPs extracts for 1, 3 and 6 days were harvested, washed and stained with Annexin-V APC (1:100) in an ice bath for 25 min. Then, each tube was added with 5 µl 7-AAD and incubated for 5 min at normal temperature in the dark. The apoptosis rate was detected by the flow cytometer (BD FACSCanto Plus, USA).

#### Cell polarization

To observe the influence of MgMPs extracts on the polarization of THP-1-derived macrophages, flow cytometry was used to quantitatively test the expression levels of the macrophage surface markers CD86 (BD, USA) and CD206 (BD, USA). After cultured with different concentrations of MgMPs extracts for 1, 3 and 6 days, the cells were trypsinized, blowed and detached from the wells. Then, the harvested cells were blocked with phosphate buffered saline (PBS) containing 1% bovine serum albumin (BSA) for 40 min, stained with CD86 and CD206 for another 40 min at normal temperature in the dark. After washed and resuspended in 1% BSA, the samples were analyzed by the flow cytometer.

### Immunofluorescence staining assays

Immunofluorescence staining were used to qualitatively test the expression levels of the macrophage surface markers CCR7 and CD206. The cells were pre-treated with different concentrations of MgMPs extracts in glass-bottom dishes (3 × 10^5^ cells per dish) for 1, 3 and 6 days. After fixed, permeabilized and blocked, the harvested cells were incubated with the primary antibodies CCR7 (AbCam, USA) and CD206 (AbCam, USA) at 1:100 and 1:500, respectively in 1% BSA/PBS for 1 h at normal temperature. Afterwards, the secondary antibodies (ZSGB-BIO, China) Alexa Fluor 594 and 488 (1:200) for CCR7 and CD206, respectively were incubated with the cells at 4°C overnight. Finally, DAPI (ZSGB-BIO, China) was used to stain the position of the cell nucleus. The cellular fluorescence distribution was imaged by the fluorescence microscope (Leica SP8, Germany).

### Real-time quantitative PCR test

The relative mRNA levels of THP-1-derived macrophages-related genes (TNF-α and IL-1β) under different conditions for the indicated durations was assayed by real-time quantitative PCR. The harvested cells were resuspended with TRIZOL solution (Invitrogen, USA) for total RNA extraction. After quantified with a micro volume spectrophotometer (Jenway, UK), 2 µL total RNA from each group was used to synthesize cDNA with a cDNA synthesis kit (Yeasen, China). Real-time PCR was carried out by Eco 48 real-time PCR detection system (PCRmax, UK). The relative mRNA expressions were calculated using ΔΔCt method and compared to the housekeeping gene GAPDH. The primers used in our research are listed in Table 1.

**Table 1:** Primers used for real-time quantitative PCR

Target gene	Direction	Sequence (5’-3’)
GAPDH	Forward	GGAGCGAGATCCCTCCAAAAT
	Reverse	GGCTGTTGTCATACTTCTCATGG
TNF-α	Forward	CGAGTCTGGGCAGGTCTA
	Reverse	GTGGTGGTCTTGTTGCTTAA
IL-1β	Forward	CCCTCTGTCATTCGCTCCC
	Reverse	CACTGCTACTTCTTGCCCCC

### Inflammatory cytokine test

The sandwich enzyme-linked immunosorbent assay (ELISA) principle was used to measure the related cytokine secretions. After treated in combination with different concentrations of MgMPs extracts, the samples were centrifuged and the supernatants were harvested. Enzyme-linked immunosorbent was conducted as described in the manufacturer’s instructions of the ELISA kits (R&D, USA). The concentrations of IL-1β, TNF-α and IL-10 were calculated based on the absorbance of the test sample and the standard curve.

### Statistical analysis

Individual experiments involving statistical analysis in this study were repeated three or more times. All the data were expressed as means ± standard deviation (SD) and performed by Prism 7 (GraphPad Software, USA). The differences among means were considered the level of significance at *P < 0.05 and **P < 0.01.

## Results and discussions

### MgMPs extracts characterization

**Figure 1:**
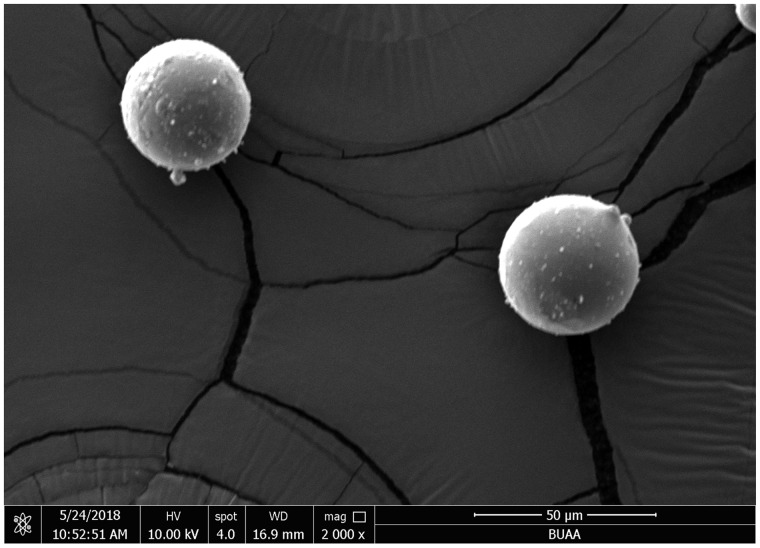
Morphology of the granulated MgMPs.

The morphology of MgMPs was characterized by SEM and the result is shown in Fig. 1. The MgMPs were spherical in shape with smooth surfaces. The particle size was <50 μm in diameter. In order to maintain fairly good cell viability, we adjusted the MgMPs extracts to pH 7.4 by adding HCl to avoid the side effect derived from the difference of pH value. The samples used in this study and the concentrations of MgMPs extracts are summarized in Table 2. With the decrease in dilution multiple, an increase in the concentration of Mg^2+^ was detected in MgMPs extracts.

**Table 2:** The physicochemical characteristics of MgMPs extracts

Samples	Mg^2+^ concentration(mg/l)	Groups
Ctr (1640 medium)	10.00	m/mono-ctr
12.5% Extract	35.22 ± 0.71	m/mono-1/8
25% Extract	70.45 ± 1.41	m/mono-1/4
50% Extract	140.90 ± 2.83	m/mono-1/2
100% Extract	281.80 ± 5.66	m/mono-1

### MgMPs extracts on the proliferation of cells

**Figure 2:**
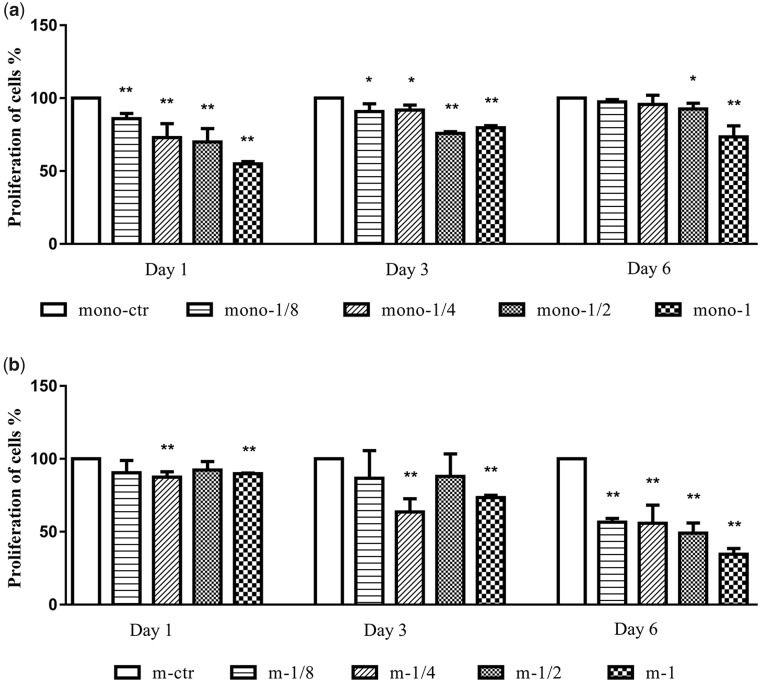
Proliferation of THP-1 cells and THP-1-derived macrophages. CCK-8 tests of (a) THP-1 cells and (**b**) THP-1-derived macrophages were assayed on Day 1, 3 and 6 in response to different Mg^2+^ concentrations or RPMI 1640. *P < 0.05 vs. mono-ctr or m-ctr group, **P < 0.01 vs. mono-ctr or m-ctr group.

In order to examine whether Mg^2+^ concentrations have effect on the proliferation of THP-1 cells and THP-1-derived macrophages, we evaluated their proliferation after they cultured in MgMPs extracts for 1-6 days. As shown in Fig. 2a, the proliferation of monocytes was hardly affected by Mg^2+^ in addition to high Mg^2+^ concentration groups (m/mono-1/2 and m/mono-1) for 6 days of treatment, even though a highly reduction over the early duration (1 and 3 days). The results showed in Fig. 2b revealed a significant reduction in macrophages proliferation starting from the m-1/4 group with Mg^2+^ concentrations of 70.45 ± 1.41 mg/l, and the trend was more obvious on Day 6. This indicated that Mg^2+^ markedly suppressed the proliferation of the macrophages as the increasing concentrations. Of note, the MgMPs extracts affected the proliferation of cells in a time and concentration-dependent manner, especially for the THIP-1-derived macrophages.

In this study, we found that high concentrations of Mg^2+^ could demote both of the two inflammatory cells on proliferation in early and late stage, respectively. It seems like THP-1 cell was more adaptive than macrophage to higher concentration of Mg^2+^ after 6 days of culture. Compared with the macrophages, the non-adhesive and hyperproliferative properties of THP-1 cells may explain this phenomenon.

### MgMPs extracts on the apoptosis of cells

**Figure 3:**
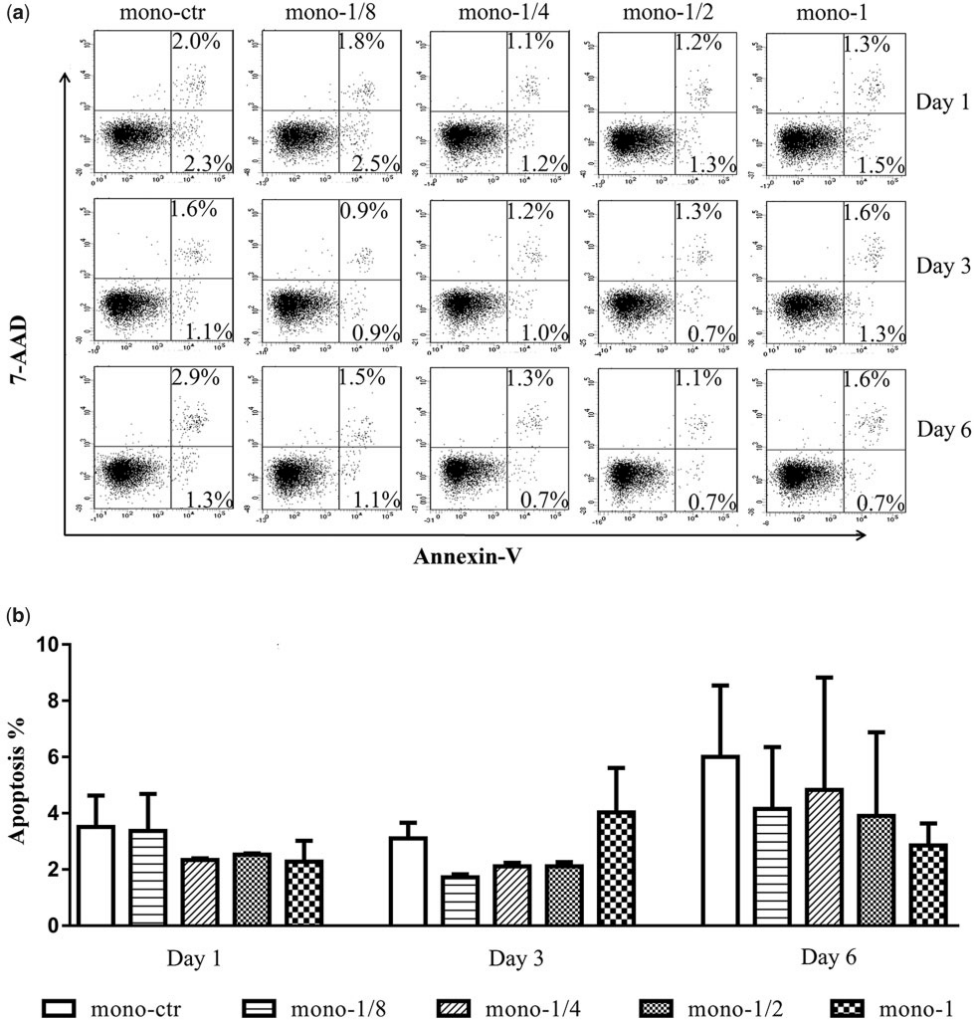
Apoptosis of THP-1 cells. (**a**) A representative result and (**b**) statistical results on death modes of THP-1 cells induced by MgMPs extracts with different Mg^2+^ concentrations or RPMI 1640, which were assayed by FACS on Day 1, 3 and 6, respectively.

**Figure 4:**
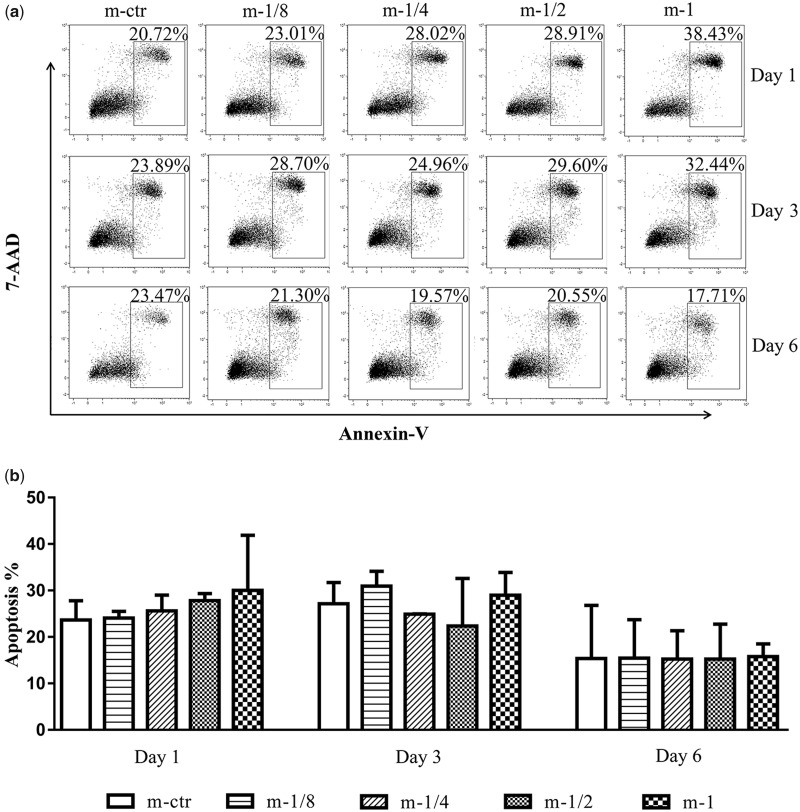
Apoptosis of THP-1-derived macrophages. (**a**) A representative result and (**b**) statistical results on death modes of THP-1-derived macrophages induced by MgMPs extracts with different Mg^2+^ concentrations or RPMI 1640, which were assayed by FACS on Day 1, 3 and 6, respectively.

The low proliferation may also be caused by altered apoptosis. In order to exam this possibility, we tested the cell viability at the same time harvested in proliferation test. As shown in Figs 3 and 4, Annexin V/PI staining revealed that although after differentiating to macrophages, the THP-1 cells had a high rate of apoptosis. However, no significant difference was found in apoptosis of these two inflammatory cells when incubated with MgMPs extracts from 1 to 6 days compared with the control group. Furthermore, when the cell culture time increased to Day 6, the THP-1 cells revealed an unstable state, while the state of macrophages gradually stabilized compared with other time of culture. The results implied that the influence of MgMPs extracts on proliferation of THP-1 cells and its derived macrophages was not due to cell apoptosis.

### MgMPs extracts on phenotype switch of THP-1-derived macrophages

Since the non-PMA treated THP-1 cells cannot be differentiated to macrophages and then polarized by Mg^2+^, the investigation of MgMPs extracts on cell polarization focused on THP-1-derived macrophages in the following study.

**Figure 5:**
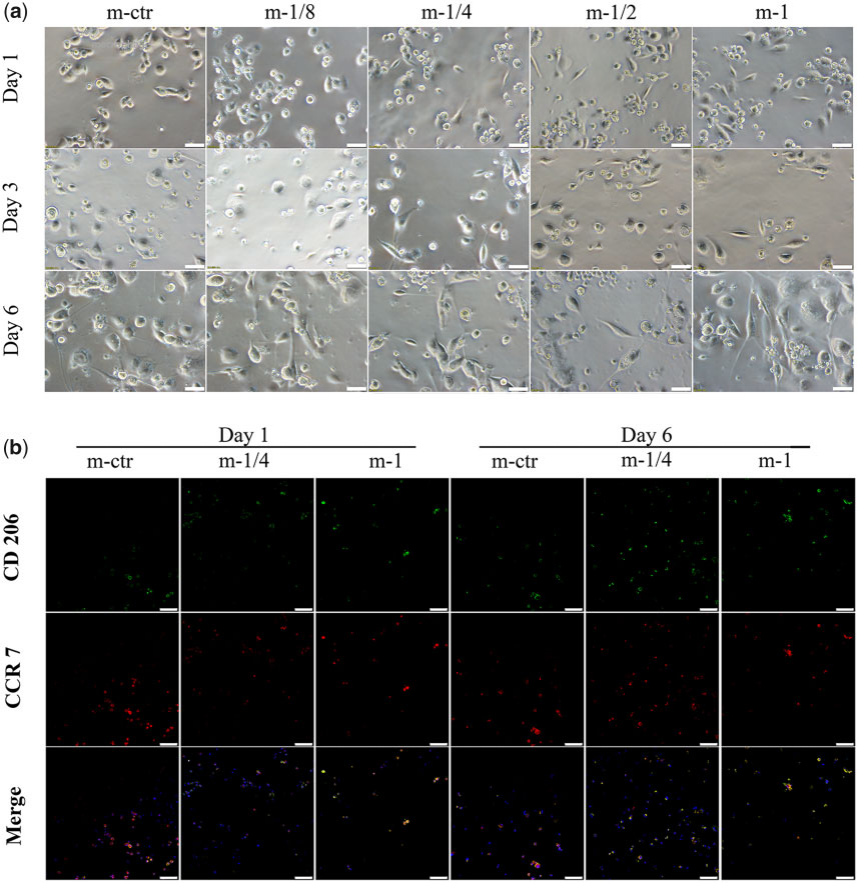
Qualitative analysis of THP-1-derived macrophage polarization. (**a**) Cytomorphology change of THP-1-derived macrophages in the presence of various MgMPs extracts on Day 1, 3 and 6, respectively. Scale bar: 50 µm. (**b**) The images of immunofluorescent staining of THP-1-derived macrophages in response to MgMPs extracts on Day 1 and 6, respectively. CCR 7 (red fluorophore) indicated M1 macrophages, CD206 (green fluorophore) indicated M2 macrophages, and nuclei were stained with DAPI (blue fluorophore). Scale bar: 75 µm.

No difference was found on the macrophage morphology between low Mg^2+^ concentration groups (m-1/8 and m-1/4) and control group after 1 day of culture. However, the numbers of macrophages decreased especially in the m-1/2 and m-1 group on Day 6, which was in accordance with the result of CCK-8 assay (Fig. 2b). Furthermore, most of the macrophages in control group were shown flat and round type with less pseudopodia, whereas the macrophages in MgMPs extracts-treated groups were shown more elongated pseudopodia as increasing Mg^2+^ concentrations or prolonging culture duration (Fig. 5a). Changes in the shape of THP-1-derived macrophages are supposed to be attributed to the polarization state after differentiation.

The influence of Mg^2+^ on macrophage phenotype switch was evaluated by immunofluorescence staining assay. Macrophages cultured in the presence of MgMPs extracts expressed less CCR7 and more CD206 than the control group and the expression amount decreased as Mg^2+^ concentrations increased. For the same Mg^2+^ concentrations groups, the amount of CCR7 also decreased as prolonging culture time, whereas the expression of CD206 was increased (Fig. 5b).

**Figure 6:**
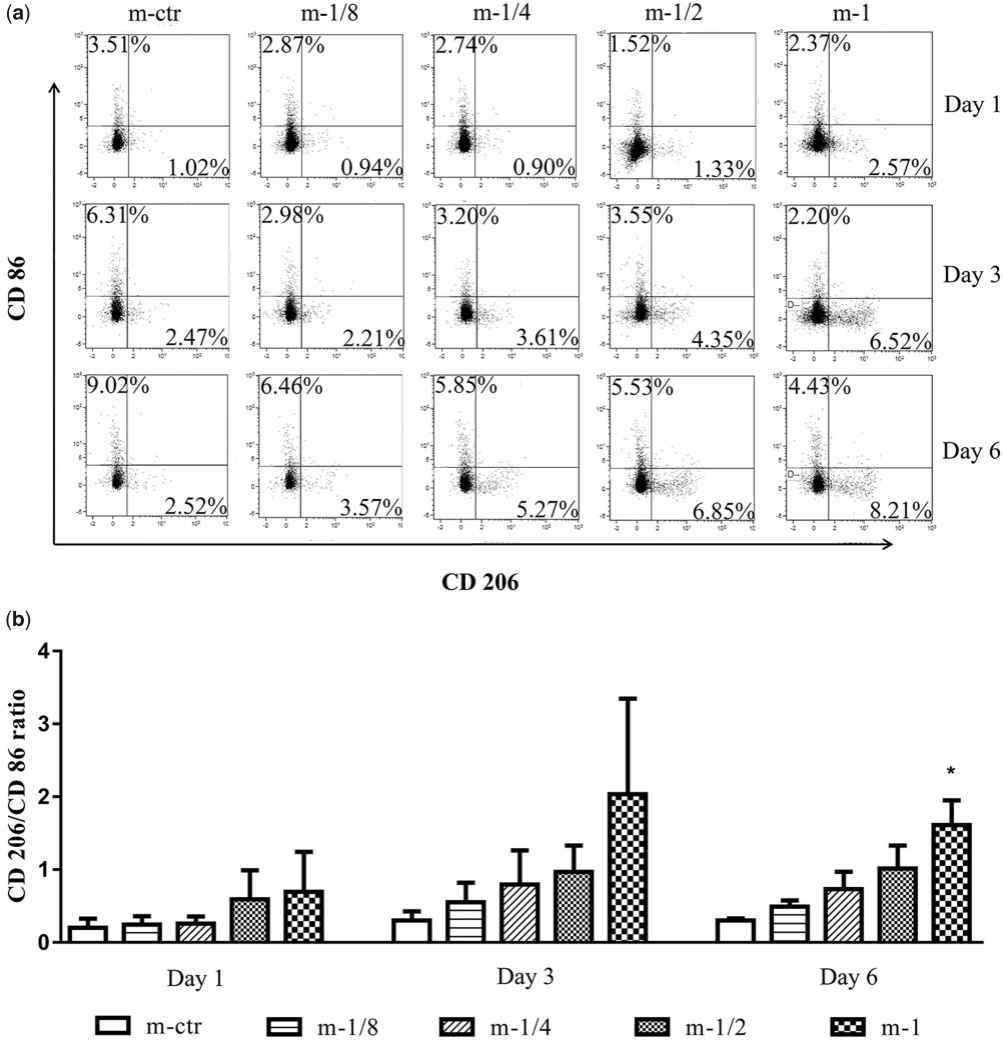
Quantitative analysis of THP-1-derived macrophage polarization. (**a**) A representative result of cell surface markers CD86 and CD206 expressions on THP-1-derived macrophages determined by flow cytometry. (**b**) The statistical analysis of CD206/CD86 ratio calculated from the flow cytometric plots of five groups at varied time points. *P < 0.05 vs. m-ctr group.

Furthermore, we double-checked the result by flow cytometry (Fig. 6a and b). Different concentrations of Mg^2+^ notably affected the percentages of CD86 and CD206 positive macrophages compared with those of the control group. The increased CD206/CD86 ratio indicated that, as an increasing concentration of Mg^2+^, the CD206 positive macrophages increased significantly, together with the reduction in CD86 positive macrophages. On Day 6, the m-1 group had almost triple percentage of CD206^+^ M2-like macrophage, while the percentage of CD 86^+^ M1-like macrophage dropped to less than half.

Briefly, both of the immunostaining and flow cytometry results have showed that the Mg^2+^-treated macrophages presented an up-regulated M2 marker and down-regulated M1 marker expression, and this trend was becoming more obvious over time.

### MgMPs extracts on inflammatory responses of THP-1-derived macrophages

**Figure 7:**
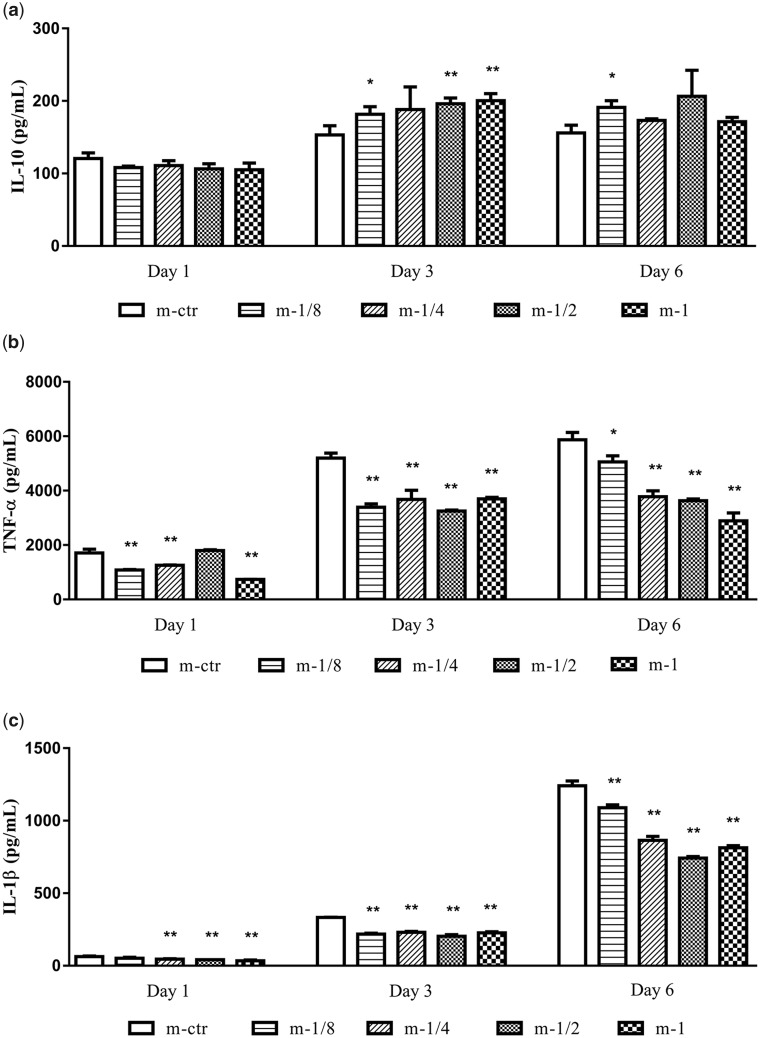
MgMPs extracts regulated inflammatory responses of THP-1-derived macrophages. Cytokines secretion of (**a**) IL-10, (**b**) TNF-α and (**c**) IL-1β in THP-1-derived macrophages treated with different concentrations of Mg^2+^ for 6 days by ELISA. RPMI 1640 treatment alone was the control group. *P < 0.05 vs. m-ctr group, **P < 0.01 vs. m-ctr group.

To further confirm the immunomodulation of Mg^2+^ on THP-1-derived macrophages, we inspected cytokines secretion and relative mRNA expression. As presented in Fig. 7, the secretion of pro-inflammatory cytokines TNF-α (Fig. 7b) and IL-1β (Fig. 7c) in response to MgMPs extracts were down-regulated compared with the control group on Day 1 and 3, even with the lowest tested Mg^2+^ concentrations. After 6 days of culture, the secretions of these two pro-inflammatory cytokines became obvious related with Mg^2+^ concentrations in a dose-dependent manner. Furthermore, the secretion of anti-inflammatory cytokine IL-10 (Fig. 7a) was not significantly influenced by the stimulation of Mg^2+^ on Day 1 in comparison with the control group, but after 3 days of culture, the release of IL-10 increased as the increasing Mg^2+^ concentration. Further prolonging culture time to Day 6, the expression level of IL-10 was similar with that on Day 3. All these results showed that MgMPs extracts suppressed the pro-inflammatory cytokines release while promoting the anti-inflammatory cytokine release.

**Figure 8:**
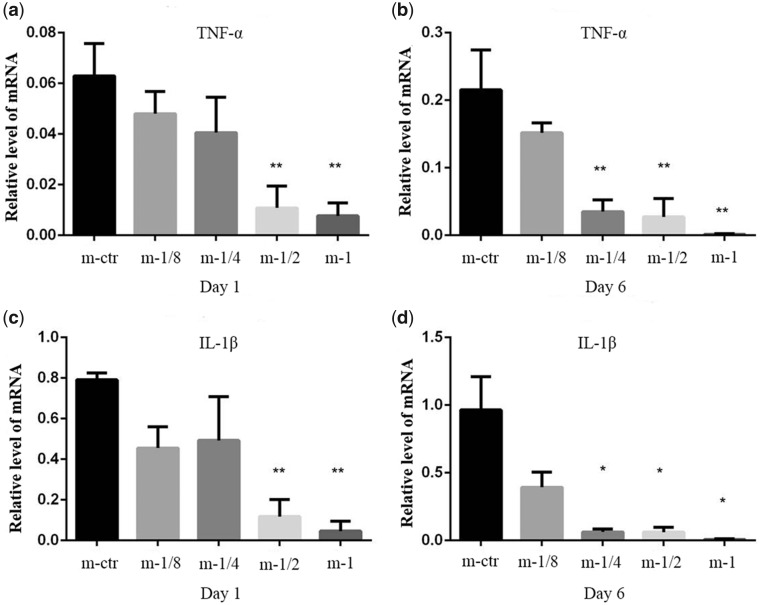
Macrophage function as determined by PCR. Relative mRNA expression of pro-inflammatory TNF-α (**a** and **b**) and IL-1β (**c** and **d**) were detected by real-time PCR assay. GAPDH was used as the housekeeping gene. RPMI 1640 treatment alone was the control group. *P < 0.05 vs. m-ctr group, **P < 0.01 vs. m-ctr group.

As shown in Fig. 8, the mRNA expressions of pro-inflammatory (TNF-α and IL-1β) exhibited a significant down-regulated trend after stimulated by Mg^2+^, and such trend became more obvious after 6 days of treatment. This result was consistent with the result obtained by ELISA.

In brief, the alterations in cytokine and genetic levels, further confirmed the role Mg^2+^ played in on macrophages switch polarization states.

Together, all these results indicated that MgMPs extracts could induce a less M1 macrophage activation and a characteristic M2 macrophage response, which demonstrated that Mg^2+^ could switch THP-1-derived macrophages from M0 to M2 phenotype in a time and concentration-dependent manner.

Mg^2+^-induced enhancement of basal IκB-α levels and impediment of NF-κB activation could reduce the TNF-α and IL-1β production. Therefore, the lack of Mg^2+^ in humans is often accompanied by inflammatory reactions. [11, 34, 35]. Takayanagi *et al*. [36] also reported that when high concentrations of Mg ions were found in the microenvironment, the activation of NF-κB would be inhibited, while the level of IκB-α would be enhanced. This reduction in inflammation due to the suppression effect of Mg^2+^ coincides with our findings. Hence, the result in our study might be attributed to the inhibition of Mg^2+^ on the NF-κB signaling pathway and this underlying mechanism could reduce the percentage of M1 macrophages, promote the polarization behavior of macrophage to M2 phenotype. L-type calcium channels, phosphoinositide 3-kinase/Akt and phosphoinositide 3-kinase β, δ and γ are also reported to be involved in Mg^2+^-induced anti-inflammation [37, 38]. A more precise molecular mechanisms of Mg^2+^ on macrophage polarization and inflammation is needed in further investigation.

## Conclusion

The introduction of neutralized Mg ions is demonstrated to transform THP-1-derived macrophage to M2 phenotype, which results in the less production of pro-inflammatory cytokine and more secretion of anti-inflammatory cytokine. Less than 70 mg/l of Mg^2+^ concentration is favorable for maximizing M2 polarization while maintaining cell viability. As a result, Mg^2+^ can serve as a new anti-inflammatory agent to suppress the excessive inflammatory reactions and Mg-based biomaterials may endow the implants with anti-inflammatory function, which providing a new insight into the development of immunomodulatory biomaterials.

## Funding

This study was financially supported by the National Natural Science Foundation of China (11872097, 31872735), Beijing Natural Science Foundation (L182017), the Fundamental Research Funds for the Central Universities (YWF-19-BJ-J-234), the 111 Project (B13003), and the International Joint Research Center of Aerospace Biotechnology and Medical Engineering, Ministry of Science and Technology of China.
